# Large Improvement of Thermoelectric Performance by Magnetism in Co‐Based Full‐Heusler Alloys

**DOI:** 10.1002/advs.202303967

**Published:** 2023-08-04

**Authors:** Zhigang Gui, Guiwen Wang, Honghui Wang, Yuqing Zhang, Yanjun Li, Xikai Wen, Yikang Li, Kunling Peng, Xiaoyuan Zhou, Jianjun Ying, Xianhui Chen

**Affiliations:** ^1^ CAS Key Laboratory of Strongly coupled Quantum Matter Physics and Department of Physics University of Science and Technology of China Hefei Anhui 230026 P. R. China; ^2^ Analytical and Testing Center Chongqing University Chongqing 401331 P. R. China; ^3^ College of Physics and Center of Quantum Materials & Devices Chongqing University Chongqing 401331 P. R. China; ^4^ Interdisciplinary Center for Fundamental and Frontier Sciences Nanjing University of Science and Technology Jiangyin Jiangsu 214443 P. R. China

**Keywords:** full heusler alloys, magnon drag, spin fluctuation, thermopower

## Abstract

Full‐Heusler alloys (fHAs) exhibit high mechanical strength with earth‐abundant elements, but their metallic properties tend to display small electron diffusion thermopower, limiting potential applications as excellent thermoelectric (TE) materials. Here, it is demonstrated that the Co‐based fHAs Co_2_ *X*Al (*X* = Ti, V, Nb) exhibit relatively high thermoelectric performance due to spin and charge coupling. Thermopower contributions from different magnetic mechanisms, including spin fluctuation and magnon drag are extracted. A significant contribution to thermopower from magnetism compared to that from electron diffusion is demonstrated. In Co_2_TiAl, the contribution to thermopower from spin fluctuation is higher than that from electron diffusion, resulting in an increment of 280 µW m^−1^ K^−2^ in the power factor value. Interestingly, the thermopower contribution from magnon drag can reach up to ‐47 µV K^−1^, which is over 2400% larger than the electron diffusion thermopower. The power factor of Co_2_TiAl can reach 4000 µW m^−1^ K^−2^ which is comparable to that of conventional semiconducting TE materials. Moreover, the corresponding figure of merit *zT* can reach ≈0.1 at room temperature, which is significantly larger than that of traditional metallic materials. The work shows a promising unconventional way to create and optimize TE materials by introducing magnetism.

## Graphical abstract

Electron diffusion thermopower is the dominant item in conventional thermoelectric materials. In magnetic ferromagnetic Co‐based full Heusler alloys, spin fluctuation and magnon drag make a great contribution to thermopower far larger than the conventional diffusion item. Magnetism is an unconventional way to improve thermoelectric performance and magnetic metallic systems with high carrier concentration can also be candidates for thermoelectric application.

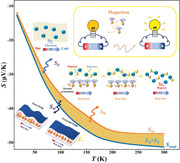

## 1 Introduction

Thermoelectric (TE) materials, which can efficiently realize the reversible conversion between heat and electricity, have attracted intensive interest in applications for power generation and refrigeration.^[^ ^1^ ^]^ The conversion efficiency of TE materials is determined by the dimensionless figure of merit *zT*, given by *zT = S^2^T/ρκ*, where *S* is the Seebeck coefficient, *ρ* is the electrical resistivity, *S^2^/ρ* is the power factor (PF), *T* is the absolute temperature, and *κ* is the sum of the electronic (*κ*_e_) and lattice (*κ*_l_) thermal conductivity. The Seebeck coefficient decreases with increasing electrical conductivity as the carrier density increases, and it is well acknowledged that the power factor can be optimized in the semiconducting region. Over the past few decades, various strategies, including band engineering^[^ ^2^ ^]^ and phonon engineering^[^ ^3^, ^4^ ^]^ have been used for the enhancement of *zT* in conventional semiconducting materials.^[^ ^5^ ^]^ Despite these efforts, the TE performance remains low due to the limited efficiency stemming from the strong coupling of basic physics parameters (*S*, *ρ*, and *κ*), which prevents its large‐scale application. According to the standard Boltzmann transport theory, enhancing both the thermopower and the electrical conductivity without increasing the thermal conductivity is a significant challenge, resulting in a dilemma in the search for ideal thermoelectric materials in conventional semiconductors. Fortunately, this predicament can be resolved through the utilization of the spin degree of freedom of electrons.^[^ ^6^, ^7^, ^8^ ^]^

Recently, magnetic mechanisms have been proven to affect the TE performance of materials^[^ ^9^, ^10^ ^]^ as an unconventional way. In a thermal nonequilibrium magnetic system, the process of transport couples electron spin and charge, leading to spin‐caloritronic^[^ ^10^ ^]^ effects such as magnon drag^[^ ^11^ ^]^ and spin fluctuation.^[^ ^12^ ^]^ Magnon drag thermopower is the chemical potential^[^ ^13^ ^]^ contribution that originates from thermally excited magnons^[^ ^14^ ^]^ interacting with electrons via magnetic scattering. For example, the semiconductor Mn_1‐x_Cr_x_Sb^[^ ^11^ ^]^ system exhibits a significant thermopower enhancement by magnon drag, with a maximum value of ≈20 µV K^−1^. Spin fluctuation, on the other hand, involves temperature‐dependent spin‐flip scattering, resulting in local magnetic excitation to enhance thermopower. With values of ≈3 µV K^−1^ and ≈250 µW m^−1^ K^−2^, a 10% thermopower and ≈20% power factor improvement have been achieved in semiconductor Fe_2_VAl systems.^[^ ^12^ ^]^ Despite providing a new method to improve TE performance, the enhancement from magnetism has shown limited or insignificant effect compared to the other traditional methods in the previous study.

**Figure 1:**
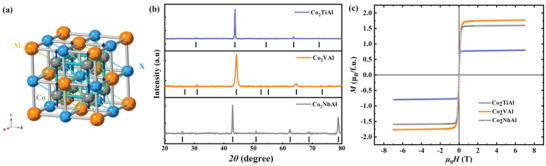
Crystal structure and M‐H curves for Co_2_ *X*Al. a) Schematic illustration of the Co_2_ *X*Al series with full‐Heusler structure. b) Room temperature powder X‐ray diffraction (XRD) patterns for Co_2_NbAl, Co_2_VAl, and Co_2_TiAl with corresponding standard PDF card (black solid line). c) Field‐dependent magnetization measurements of Co_2_NbAl at 2 K, Co_2_VAl at 80 K and Co_2_TiAl at 1.8 K.

Full‐Heusler alloys (fHAs), with the stoichiometric composition *X_2_YZ*, crystallize in a cubic structure with four interpenetrating face‐centered cubic (fcc) sublattices as shown in **Figure** **1**a. The fHAs composed of inexpensive, nontoxic, and earth‐abundant elements which exhibit high mechanical strength, making them as suitable candidates for potential thermoelectric material applications. Compared to traditional nonmagnetic metals with small thermopower and high conductivity, fHAs exhibit a variety of magnetic properties, such as ferromagnetism,^[^ ^15^, ^16^ ^]^ ferrimagnetism,^[^ ^17^, ^18^ ^]^ and anti‐ferromagnetism,^[^ ^19^, ^20^ ^]^ which provides ideal platforms to study magneto TE performance. Here, we study the thermoelectric transport properties of Co‐based fHAs, in which both *ρ* and *S* show distinct changes near the ferromagnetic transition temperatures. We discovered that the magnon drag thermopower is several tens of times larger than the conventional diffusion thermopower and the thermopower from spin fluctuation is comparable to the electron diffusion term, which results in the TE performance of Co‐based fHAs being much better than the conventional metals. The power factor of Co_2_TiAl can reach 4000 µW m^−1^ K^−2^ which is comparable to that of conventional semiconducting TE materials .^[^ ^21^, ^22^ ^]^ The corresponding figure of merit *zT* can reach ≈0.1 at room temperature, which is significantly larger than that of traditional metallic materials. Our work demonstrates that magnetic metals can be potential TE materials.

## 2 Results and Discussion

### 2.1 XRD Patterns and Magnetic Properties of Co_2_ *X*Al Series Alloy

The room temperature X‐ray diffraction (XRD) patterns for Co_2_ *X*Al (*X* = Ti, V, Nb) samples are shown in Figure 1b. All the Bragg diffraction peaks of the samples are consistent with the corresponding PDF cards, verifying that the samples were synthesized without impurity phases. The absence of some peaks indicates structural defects in the Co_2_TiAl and Co_2_VAl samples, which is common for Heusler materials.^[^ ^23^ ^]^ In addition, the diffraction pattern of Co_2_VAl in Figure 1b shows relatively broad peaks, which can be attributed to the lower crystallinity.^[^ ^24^ ^]^

Figure 1c shows the magnetization curves *M(H)* for Co_2_TiAl at 1.8 K, Co_2_VAl at 80 K and Co_2_NbAl at 2 K. The magnetization curves for all the samples show saturation behavior with a large external field and exhibit the behavior of typical ferromagnetic materials which is consistent with previous reports.^[^ ^25^, ^26^ ^]^ The temperature dependence of magnetization curves *M(T)* measured at 1000 Oe are shown in Figure S1 (Supporting Information). We can extract the Curie temperatures *T*_c_ to be 133, 337, and 393 K for Co_2_TiAl, Co_2_VAl, and Co_2_NbAl, respectively.

### 2.2 Thermoelectric Performance of Co_2_ *X*Al

**Figure 2:**
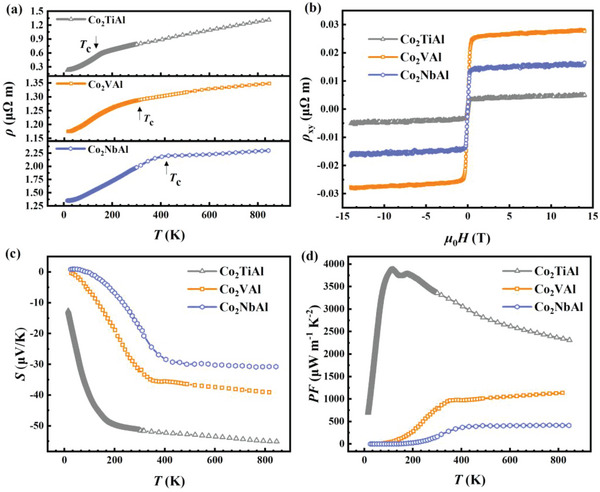
Electrical transport properties. a) Temperature‐dependent resistivity *ρ* of Co_2_TiAl, Co_2_VAl, and Co_2_NbAl at zero field. b) Isothermal Hall resistivity versus magnetic field at 100 K. c) Temperature‐dependent Seebeck coefficient *S* of Co_2_TiAl, Co_2_VAl, and Co_2_NbAl. d) Temperature‐dependent power factor PF of Co_2_TiAl, Co_2_VAl, and Co_2_NbAl.

**Figure** **2**a displays the temperature dependence of electrical resistivity (*ρ*). All the samples exhibit typical metallic behavior. The slope of *ρ(T)* suddenly changes close to the Curie temperature *T*_c_, possibly due to the change of the scattering rate and band structure^[^ ^23^, ^27^ ^]^ from the ferromagnetic (FM) state to the paramagnetic (PM) state, which is common in ferromagnetic materials.^[^ ^28^ ^]^

The Hall resistivity measured at 100 K is shown in Figure 2b. Since all the samples are in the ferromagnetic state at 100 K, they exhibit a typical anomalous Hall effect (see Figure S2, Supporting Information). The charge carrier concentration can be deduced to be 6.79×10^22^ cm^−3^ for Co_2_TiAl, 3.81×10^22^ cm^−3^ for Co_2_VAl, and 4.94×10^22^ cm^−3^ for Co_2_NbAl by calculating the Hall coefficient in the high field linear region. The corresponding mobility can be deduced to be 2.24 cm^2^ V^−1^ s^−1^ for Co_2_TiAl, 1.35 cm^2^ V^−1^ s^−1^ for Co_2_VAl and 0.85 cm^2^ V^−1^ s^−1^ for Co_2_NbAl.

The temperature‐dependent Seebeck coefficient is shown in Figure 2c. The absolute value of the Seebeck coefficient increases significantly from 20 K up to *T*_c_ and slowly increases with increasing the temperature above *T*_c_. The maximum Seebeck coefficient can reach several tens of *µV K^−1^*, which is much higher than that of traditional metals.^[^ ^29^ ^]^ Such a large Seebeck coefficient and its anomalous temperature‐dependent behavior can be attributed to the magnetic mechanism concerning magnon drag and spin fluctuation as discussed later.

Combining resistivity with Seebeck coefficient, the temperature‐dependent power factors PF were obtained, as shown in Figure 2d. For the Co_2_TiAl sample, PF increases rapidly with increasing temperature before *T*_c_ due to the rapid growth of *S*, reaching a maximum value of ≈3880 µW m^−1^ K^−1^ around *T*_c_. The PF starts to decrease slowly with increasing the temperature at high temperature due to the relatively weak temperature dependence of the Seebeck coefficient and increasing resistivity. Such power factor of Co_2_TiAl is comparable to that of some traditional semiconducting thermoelectric materials.^[^ ^30^ ^]^ In the Co_2_VAl and Co_2_NbAl samples, PF can reach relatively smaller values of ≈950 µW m^−1^ K^−1^ and ≈350 µW m^−1^ K^−1^ around *T*_c_ respectively because of the lower Seebeck coefficient and relatively large resistivity, which slowly increase with temperature due to the slow growth of the Seebeck coefficient and resistivity above *T*_c_ as shown in Figure 2a,c.

**Figure 3:**
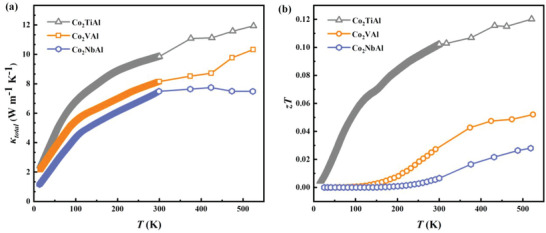
Kappa and *zT*. a) Temperature‐dependent thermal conductivity *κ*_total_ of Co_2_TiAl, Co_2_VAl, and Co_2_NbAl. b) Temperature‐dependent figure of merit *zT* of Co_2_TiAl, Co_2_VAl, and Co_2_NbAl.

The thermal conductivity *κ*_total_ of Co_2_ *X*Al is displayed in **Figure** **3**a. *κ*_total_ increases with increasing the temperature due to the large contribution of electronic thermal conductivity *κ*_e_ *_,_* as shown in Figure S3 (Supporting Information). In the low temperature region, *κ*_total_ increases rapidly with temperature below 100 K. Above 100 K, *κ*_total_ increases almost linearly with a smaller slope, where the increment of *κ*_total_ is dominated by the linear increment of electronic thermal conductivity *κ*_e_ *_,_* as shown in Figure S3 (Supporting Information). The maximum *κ*_total_ in these samples can reach approximately 10 W K^−1^ m^−1^ at room temperature, which is considerably lower than the values known for traditional metals.^[^ ^31^ ^]^ Above 300 K, we acquire *κ*_total_ according to *κ*_total_ *= ρC*_p_ *D* from the measurements of isobaric capacity *C*_p_ and thermal diffusion coefficient *D*. *κ*_total_ increases slowly with temperature due to the inverse relationship between lattice thermal conductivity and temperature, while the proportional relationship for *κ*_e_, i.e., *κ*_l_ ∝*1/T* and *κ*_e_ ∝*T*.

We can calculate the temperature‐dependent thermoelectric figure of merit *zT* as shown in Figure 3b. Co_2_TiAl has the maximum *zT* among the three samples with a value of *zT* over 0.1 at room temperature, while the value of *zT* is ≈0.03 for Co_2_VAl and 0.007 for Co_2_NbAl. Above 300 K, all the samples show a growing trend of *zT*. The value of *zT* for Co_2_VAl and Co_2_NbAl above 500 K can reach ≈0.05 an ≈0.025, respectively, and Co_2_TiAl shows considerable enhancement of *zT* with a value of ≈0.12, which is much higher than that of traditional metals.

### 2.3 Spin Fluctuation Contribution in *S*

**Figure 4:**
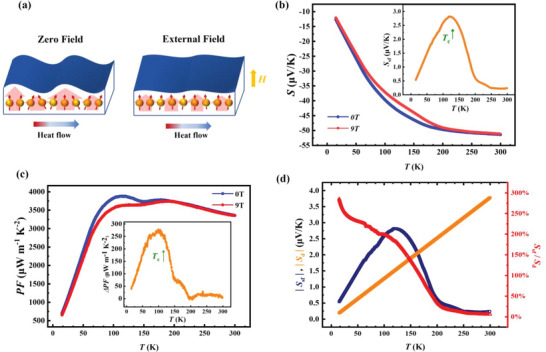
Thermoelectric contribution from spin fluctuation. a) Schematic illustration of spin fluctuation with zero field and under an external field. b) Temperature‐dependent Seebeck coefficient *S* measured under 0 and 9 T for Co_2_TiAl. The inset displays the difference in thermopower |Δ*S*| = |*S*_0T_‐*S*_9T_| between 0 and 9 T, which is considered from spin fluctuation, named *S*_sf_. c) Temperature‐dependent power factor PF measured under 0 and 9 T. The inset shows the difference in PF. d) The absolute Seebeck coefficient contribution from spin fluctuation and electron diffusion |*S*_sf_ |, |*S*_d_|, and the ratio between *S*_sf_ and the electron diffusion term *S*_d_.

The large Seebeck coefficient in Co_2_ *X*Al is rather unusual, which is possibly related to the magnetism in these samples. We first checked the spin fluctuation contribution to the Seebeck coefficient. **Figure** **4**a shows a schematic illustration of spin fluctuation. Heat flow causes thermal perturbation to spins, resulting in thermal fluctuation of spins. At low temperature, all the magnetic moments tend to be oriented in the same direction, which results in weak spin fluctuation. With increasing temperature (< *T*_c_), magnons are excited by thermal disturbance, and the correlation length of spin fluctuation increases.^[^ ^13^ ^]^ Above the Curie temperature *T*_c_, magnons get damped and the correlation length gradually decreases. Thus, spin fluctuation mainly occurs around *T*_c_. When a strong external magnetic field is applied, spin fluctuations can be suppressed, allowing us to isolate the Seebeck coefficient which is attributed by spin fluctuation. The Seebeck coefficient of Co_2_TiAl was measured at 0 and 9 T, and the difference in the Seebeck coefficient *∆S* between the two magnetic fields can be considered as spin fluctuation contribution to thermopower *S*_sf_.

Figure 4b displays the temperature‐dependent Seebeck coefficient *S(T)* of Co_2_TiAl under 0 and 9 T. The absolute values of both *S(T)* curves exhibit a steep slope change around *T*_c_. Around *T*_c_, the absolute value of *S* evidently decreases under a strong external magnetic field. The inset of Figure 4b shows the extracted *S*_sf_. It is obvious that the thermopower *S*_sf_ stemming from spin fluctuation reaches a maximum value of 2.8 µV K^−1^ near *T*_c_. The field‐dependent *S* measured at 80, 130, and 180 K are showed in Figure S5 (Supporting Information) coincident with *S*(T), which shows suppression by magnetic field with the largest suppression at the 130 K close to *T*_c_. Although the resistivity clearly decreases under the magnetic field of 9 T around *T*_c_ as shown in Figure S6 (Supporting Information), the power factor still decreases obviously as shown in Figure 4c. Such behavior indicates the great importance of thermopower *S*_sf_ in this material. The difference between the power factors with two magnetic fields, as shown in the inset of Figure 4c, illustrates the suppression effect of the magnetic field across a wide range of temperatures around *T*_c_. This effect causes a difference in the power factor up to 280 µW m^−1^ K^−2^, ≈8% of the maximum value at 0 T.

Based on the difference in the Seebeck coefficient under different magnetic fields, it is worthwhile to compare the Seebeck coefficient contribution between spin fluctuation *S*_sf_ and electron diffusion *S*_d_. In general, *S*_d_ dominates the thermopower in conventional metallic systems, and *S*_d_ is proportional to the temperature. We can acquire *S*_d_ by fitting the Seebeck coefficient under 9 T above 240 K because in this condition the linear increment of the Seebeck coefficient is mainly contributed by electron diffusion. The fitting result is reasonable since *S*_d_ is consistent with the calculated values according to the expression: $S_{d}=\frac{2}{3}{\left(\frac{\pi }{3}\right)}^{2/3}\frac{k_{B}}{e}\frac{m*}{\hbar^{2}}\frac{k_{B}T}{n^{2/3}}$ (see Figure S8, Supporting Information) ,^[^ ^32^ ^]^ where *k*_B_ is the Boltzmann constant, *e* is the electron charge, ℏ is the reduced Planck constant, m* is the effective mass and *n* is the carrier concentration. *S*_sf_ is larger than *S*_d_ until *T*_c_ as shown in Figure 4d. Although *S*_sf_ is just <3 *µV K^−1^*, the maximum ratio of *S*_sf_ /*S*_d_ is ≈300%, indicating that the Seebeck coefficient contributed by spin fluctuation is significant in such a FM metallic system.

### 2.4 Huge Thermopower Contribution Originating from the Magnon Drag Effect

**Figure 5:**
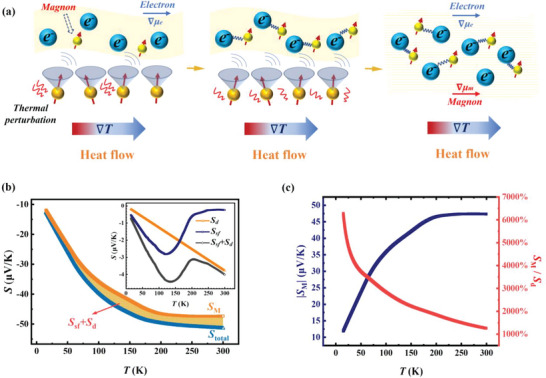
Thermopower contribution from magnon electron drag. a) Schematic illustration of magnon drag thermopower. b) Measured thermopower *S*_total_ and magnon drag induced thermopower *S*_M_ for Co_2_TiAl. The area between the *S*_total_ and *S*_M_ lines represents the sum of thermopower from spin fluctuation and electron diffusion. The inset intuitively displays the temperature‐dependent thermopower of *S*_sf_ *+S*_d_ and their respective values. c) The blue line represents the absolute thermopower of magnon drag thermopower |*S*_M_ |, and the red line represents the ratio of *S*_M_ and *S*_d_.

Besides the spin fluctuation contribution to thermopower, the magnon drag mechanism plays a crucial role in enhancing thermopower. As **Figure** **5**a shows, when heating one side of the sample and cooling the other side, a heat flow is generated, creating a gradient of temperature ∇*T*. This temperature gradient causes electrons to drift along the gradient, resulting in a gradient of chemical potential ∇µ_e_^[^ ^13^ ^]^ for the diffusion thermopower item *S*_d_=  ∇µ_e_/∇*T*. This is regarded as the traditional mechanism thermopower. In magnetic materials, the heat flow along the temperature gradient will cause thermal perturbation to spins, and therefore, magnons can be excited. The drifted electrons may interact with those excited magnons through a spin‐conserving scattering process, resulting in a drag effect on electrons. This drag force makes another contribution to thermopower, which is called magnon‐electron drag thermopower *S*_M_, with the corresponding gradient of chemical potential labeled ∇µ_m_, i.e., *S*_M_=  ∇µ_m_/∇*T*. Magnons and electrons can now be modeled as two interpenetrating fluids,^[^ ^32^ ^]^ therefore, such thermopower consists of two components: the electron diffusion thermopower *S*_d_ and magnon drag thermopower *S*_M_.

By subtracting the diffusion thermopower and spin fluctuation item, the magnon drag thermopower of Co_2_TiAl is plotted with the label *S*_M_ as shown in Figure 5b. The area between *S*_total_ and *S*_M_ is the contribution of both spin fluctuation and electron diffusion, which is shown intuitively in the inset of Figure 5b. To accurately analyze the thermopower in magnetic materials, it is necessary to distinguish the state of magnetic order with temperature, i.e., FM state below *T*_c_ and PM state above *T*_c_. In the FM state, *S*_total_ rapidly increases as the temperature approaches *T*_c_. As shown in Figure 5b, although the contribution of the spin fluctuation effect discussed before is deducted, the thermopower *S*_M_ still increases rapidly with temperature. Above *T*_c_, the *S*_M_ remains large, which implies that the magnetic mechanism continues to have an impact on the thermopower in the PM state. Some evidence proves that short range magnetic order may exist above the transition temperature by means of neutron scattering,^[^ ^13^ ^]^ which may extend the magnon drag effect to the paramagnetic region, labeled as paramagnon drag.^[^ ^13^ ^]^

In order to present thermopower contribution from magnon drag more clearly and intuitively in Co_2_TiAl, the absolute value of the difference and the ratio of thermopower between *S*_M_ and *S*_d_ are shown in Figure 5c. Because of the low diffusion thermopower at low temperature, the ratio of thermopower (*S*_M_/*S*_d_) is over 6000%. Moreover, the ratio remains at a high value of ≈2400% around *T*_c_. Importantly, the magnon drag thermopower is over 10 times larger than the electron diffusion thermopower at room temperature. Such a high ratio of *S*_M_/*S*_d_ reflects the huge thermopower contribution from the magnon drag effect. As a metallic system, Co_2_TiAl holds the largest thermopower contribution from the magnetic mechanism compared to the traditional diffusion term in most TE materials.

## 3 Discussion

Since multiple magnetic mechanisms work together in Co2XAl system to improve thermoelectric performance, it is necessary to have further discussion and analysis to distinguish the enhancement from different mechanisms.

Regarding the difference in Seebeck coefficient (∆S) and power factor (∆PF) between 0 and 9 T, as discussed in section 2.3, it is likely that such differences arise from spin fluctuation. Both *∆*S and *∆*PF reach their maximum values around *T*_c_, which is the typical behavior of spin fluctuation in consistent with the other relevant results reported previously.^[^ ^12^, ^33^ ^]^ In addition, the extracted thermopower from spin fluctuation in our work can be fitted well with theoretical model^[^ ^34^ ^]^ that describes spin fluctuation thermopower in ferromagnetic systems(See Figure S7, Supporting Information). Based on experimental behavior and fitting result, it is convincing that spin fluctuation contributes to the thermopower. Besides this, another magnetic mechanism should be taken into consideration, spin entropy, which also shows the suppression effect in magnetic field similar to spin fluctuation. In fact, spin entropy exhibits different response to magnetic field compared to spin fluctuation. For spin fluctuation in FM systems, the suppression of thermopower under magnetic field increases from zero temperature to magnetic transition temperature *T*_c_ and reaches the maximum value near *T*_c_, and then decreases above *T*_c_. For spin entropy in FM systems or AFM systems, the suppression of thermopower under magnetic field is small below the magnetic transition temperature *T*_c_ or *T*_N_ and starts to grow up quickly above *T*_c_ or *T*_N_, and finally reaches the maximum value at a certain temperature above *T*_c_/*T*_N_.^[^ ^8^, ^35^, ^36^ ^]^ Based on this, such enhancement in our work tends to be dominated by spin fluctuation.

Furthermore, the huge enhancement of thermopower discussed in section 2.4 displays different behavior compared to spin fluctuation, which is regarded as the contribution from magnon drag. According to the Hydrodynamic theory,^[^ ^32^ ^]^ magnon drag thermopower can be modeled as $S_{M}\;=\;\frac{2}{3}\frac{C_{m}}{n_{e}e}\frac{1}{1\;+\;\tau_{\mathrm{em}}/\tau_{m}}$, where *C*_m_ is the magnon specific heat capacity, *n*_e_ is the carrier density, *e* is the electron charge, *τ*_m_ is the relaxation time of magnons and *τ*_em_ is the relaxation time reflecting magnon‐electron interaction. If regarding *τ*_m_ and *τ*_em_ as constants, magnon drag thermopower is proportional to *C*_m_, which exhibits exponential temperature dependence *T^n^*. Under this condition, *S*_M_ in ferromagnetic systems follows *T* ^3/2^ law and in antiferromagnetic systems follows *T* ^3^ law. The magnon drag thermopower of all three samples were plotted with log‐log scale in Figure S9 (Supporting Information), and the fitting results show *n*≈1 for Co_2_TiAl, *n*≈1.5 for Co_2_VAl and *n*≈1.7 for Co_2_NbAl which is close to *n*≈1.5 in ferromagnetic systems. The deviation of *n* for Co_2_TiAl can be attributed by τ_em_, which is inappropriate to be treat as a constant since the Co_2_TiAl exhibits relative disorder compared to other two samples as discussed later.

Another question which needs to be considered is why Co_2_TiAl exhibits a higher enhancement in TE performance from magnetism than Co_2_VAl and Co_2_NbAl. First, it is important to note that although they have typical fHAs crystal structures, there is disorder among Ti and Al atoms in Co_2_TiAl, while Co_2_NbAl is highly ordered. Such disorder may probably influence the relaxation time for electron‐magnon interactions^[^ ^32^ ^]^ which is related to the magnon drag effect. For Co_2_VAl, the lower crystallinity may hinder the enhancement of TE performance by magnetism. Second, the effective magnetic moment *µ*_eff_ should also be taken into account. The three samples exhibit different *µ*_eff_ values of 0.79 *µ*_B_ for Co_2_TiAl, 1.82 *µ*_B_ for Co_2_VAl and 1.62 *µ*_B_ for Co_2_NbAl. Combining our results on Co_2_TiAl and the thermopower contribution from magnetism in Co_2_VAl and Co_2_NbAl (see Figure S10, Supporting Information), it seems that there is no direct relation between the magnitude of the effective magnetic moment and the enhancement of thermopower. Further experiments are needed to unveil the effect of *µ*_eff_ on the enhancement of TE performance in these materials.

More importantly, it is worthwhile to discuss how to properly enhance the TE performance by magnetism in TE materials. Based on our results and Hydrodynamic theory,^[^ ^32^, ^37^ ^]^ magnon drag thermopower is proportional to (1+ *τ*_em_ */τ*_m_)^−1^ where *τ*_em_ and *τ*_m_ are the relaxation time for electron‐magnon and magnon‐magnon. Disorder might indirectly cause the reduction of *τ*_em_ to enhance magnon drag thermopower. Spin motive force theory^[^ ^32^, ^38^, ^39^ ^]^ for magnon drag thermopower gives a more practical way compared to the former one to guide us to boost magnon drag thermopower. Such theory attributes magnon drag thermopower to spin‐orbit interactions and corresponding current can be regarded to be pumped by dynamic magnetization by spin‐motive forces. It is reported that *S*_M_ might be proportional to the ratio^[^ ^12^ ^]^ *M*_t_ /*M*_i_, where *M*_t_ is the total moment of spin and *M*_i_ is the itinerant moment of spin. This implies that a system with higher *M*_t_ but lower *M*_i_ can promisingly generate a huge magnon drag thermopower. For spin fluctuation, the contribution to thermopower is studied by two‐band model^[^ ^40^ ^]^ theoretically showing the relevance between *d* band of electrons and spin fluctuation. The results show that *d* electrons contribute more than *s* electrons, indicating heavy *d* band of electrons can account for the relatively strong spin fluctuation. It can be a guideline to enhance the spin fluctuation induced thermopower from electronic structure.

## 4 Conclusion

In summary, our work demonstrates the remarkable thermopower contribution from spin fluctuation and magnon drag in FM Co_2_ *X*Al. The spin fluctuation contribution to the thermopower of Co_2_ *X*Al is comparable to the electron diffusion term, and the thermopower from magnon drag is tens of times larger than *S*_d_ below *T*_c_. Co_2_TiAl shows excellent thermoelectric performance among Co_2_ *X*Al series fHAs, and the figure of merit *zT* can reach ≈0.1 at room temperature, which is considerably larger than that of traditional metals. Our work indicates that the magnetic mechanism can effectively enhance the TE performance, which provides a new and promising route to explore and optimize the TE performance by introducing magnetism into TE materials.

## 5 Experimental Section

Co_2_ *X*Al series alloys were synthesized by the arc‐melted method.^[^ ^25^, ^26^, ^41^ ^]^ The as‐cast ingots were annealed at 1123 K for 7 days followed by furnace cooling. Powder X‐ray diffraction data were collected at room temperature using an X‐ray diffractometer (SmartLab‐9, Rikagu) with Cu *K*_α_ radiation and a fixed graphite monochromator. Magnetization measurements were performed using a Quantum Design VSM system. Resistivity and Hall resistivity were measured using the standard four‐probe method in a Quantum Design PPMS‐9T system. The thermopower and kappa of Co_2_VAl and Co_2_NbAl below 300 K were measured with a home‐built setup by applying a steady heat current through the sample (steady‐state method) in a cryostat, and the thermopower and kappa of Co_2_TiAl below 300 K was measured with a home‐built setup by applying an alternating heat current through the sample (AC method) in a Quantum Design PPMS‐9T system. The resistivity and thermopower above 300 K were measured using a Cryoall CTA‐3 system by the steady‐state method. The thermal conductivity above 300 K was calculated by the equation κ  =  *DC*_p_ *ρ*, where *D* is the thermal diffusivity measured by the laser flash method (LFA 467, Netzsch) and *C*_p_ is the isobaric heat capacity obtained on a differential scanning calorimeter (Netzsch 404 F3).

## Conflict of Interest

The authors declare no conflict of interest.

## Supporting information

Supporting Information

## Data Availability

The data that support the findings of this study are available from the corresponding author upon reasonable request.
