# Supplementary material for: Large Improvement of Thermoelectric Performance by Magnetism in Co‐Based Full‐Heusler Alloys
Source: Adv Sci (Weinh). 2023 Aug 4;10(28):2303967. doi: 10.1002/advs.202303967 (PMC10558654; doi:10.1002/advs.202303967)
Supplement: Supplementary file 1 — Supporting Information [file ADVS-10-2303967-s001.pdf]

ADVANCED SCIENCE
Open Access

Supporting Information

for *Adv. Sci.*, DOI 10.1002/advs.202303967

Large Improvement of Thermoelectric Performance by Magnetism in Co-Based Full-Heusler Alloys

Zhigang Gui, Guiwen Wang, Honghui Wang, Yuqing Zhang, Yanjun Li, Xikai Wen, Yikang Li, Kunling Peng, Xiaoyuan Zhou, Jianjun Ying and Xianhui Chen**

Supporting Information for

Large improvement of thermoelectric performance by magnetism in Co-based full-Heusler alloys

Part I. Magnetism.

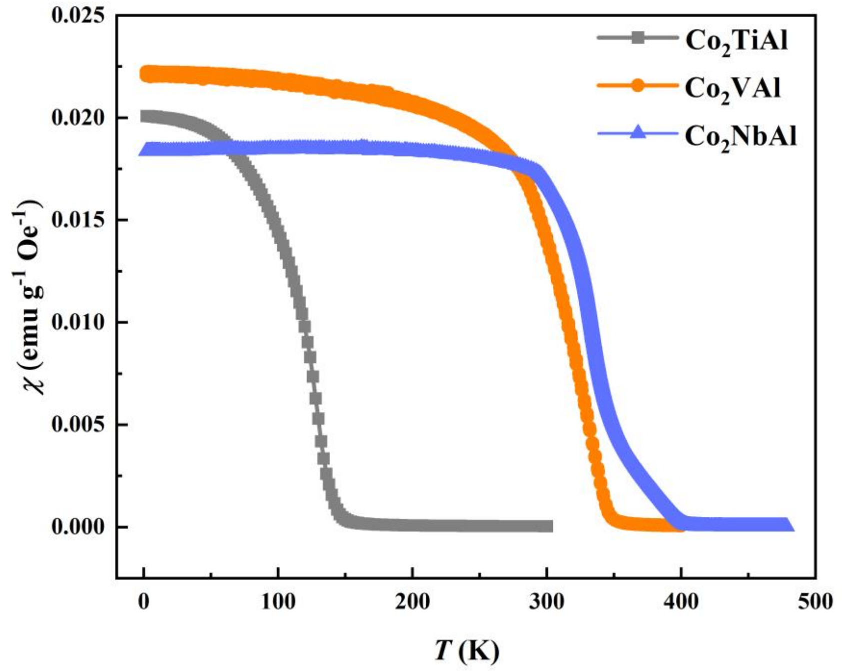

S1. Temperature-dependent magnetic susceptibility curves χ-*T*.

Figure S1 exhibits the temperature-dependent susceptibility measured under a 1000 Oe magnetic field. The gray line, orange line and blue line represent Co_2TiAl, Co_2VAl, Co_2NbAl, respectively.

Part II. Anomalous Hall conductivity.

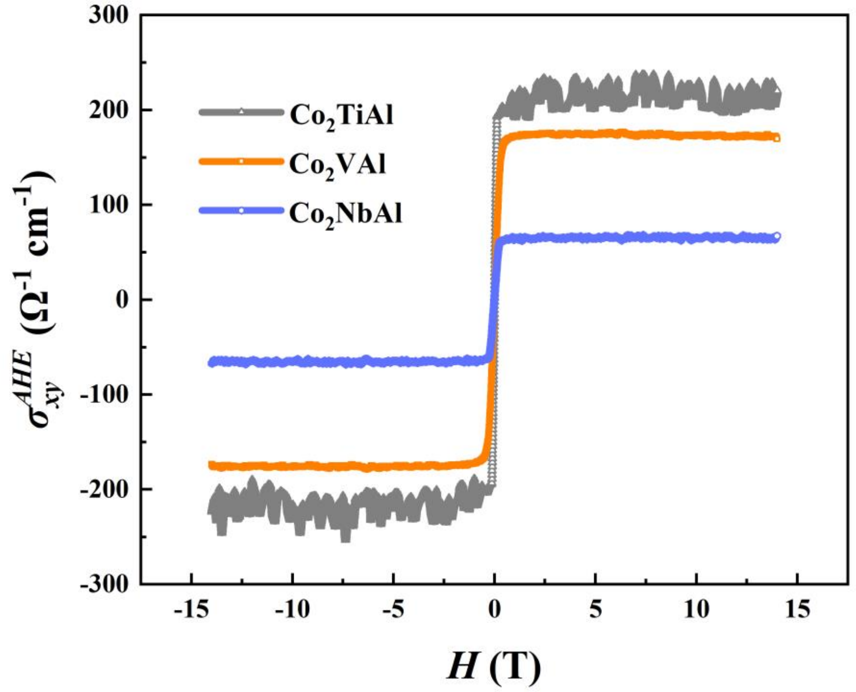

S2. Anomalous Hall conductivity of Co_2TiAl, Co_2VAl and Co_2NbAl.

Figure S2 exhibits anomalous Hall conductivity σ_{xy}^{AHE}, calculated by longitudinal resistivity ρ_{xx} and anomalous Hall resistivity ρ_{xy}^{AHE}, which is determined by extracting the linear ordinary Hall resistivity. Co_2TiAl shows a relatively large anomalous Hall conductivity $\sigma_{xy}^{AHE} \approx 220\ \Omega^{-1}cm^{-1}$, while $\sigma_{xy}^{AHE} \approx 170\ \Omega^{-1}cm^{-1}$ for Co_2VAl and $\sigma_{xy}^{AHE} \approx 65\ \Omega^{-1}cm^{-1}$ for Co_2NbAl.

Part III. Thermal conductivity.

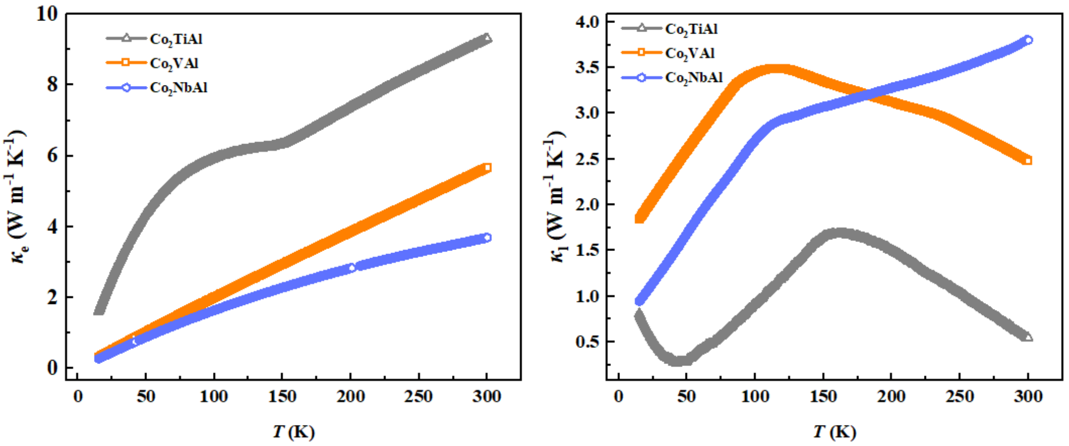

S3. Temperature-dependent electronic and lattice thermoconductivity κ_e and κ_l.

Figure S3 shows the temperature-dependent electronic and lattice thermoconductivity κ_e and κ_l of Co_2TiAl, Co_2VAl, and Co_2NbAl. κ_e is determined by the Wiedemann-Franz law, $\kappa_e = L\sigma T$, where L is the Lorentz constant, T is the absolute temperature, and σ is the electrical conductivity based on resistivity measurements. Lattice thermal conductivity is calculated from κ_{total} and κ_e.

Part IV. Thermal measurements above 350 K.

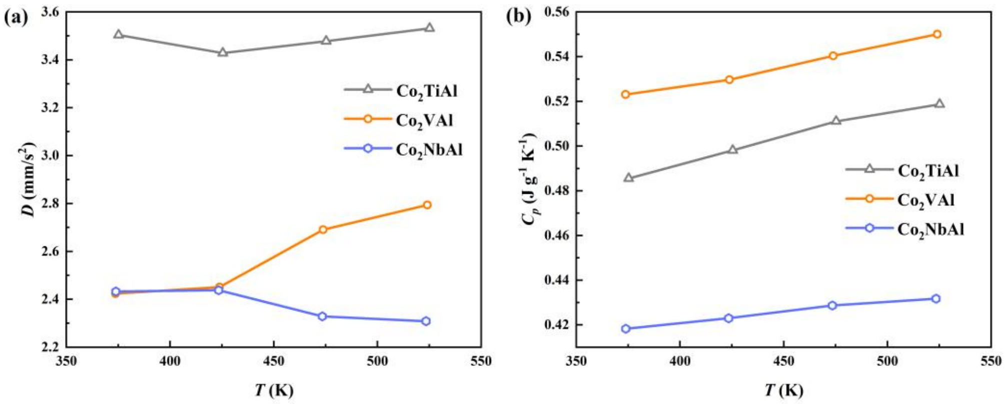

S4. Temperature-dependent diffusion coefficient and isobaric capacity above 350 K.

Figure S4 shows the temperature-dependent thermal diffusion coefficient D and isobaric capacity C_p above 350 K of Co_2TiAl, Co_2VAl and Co_2NbAl.

Part V. Field-dependent thermopower.

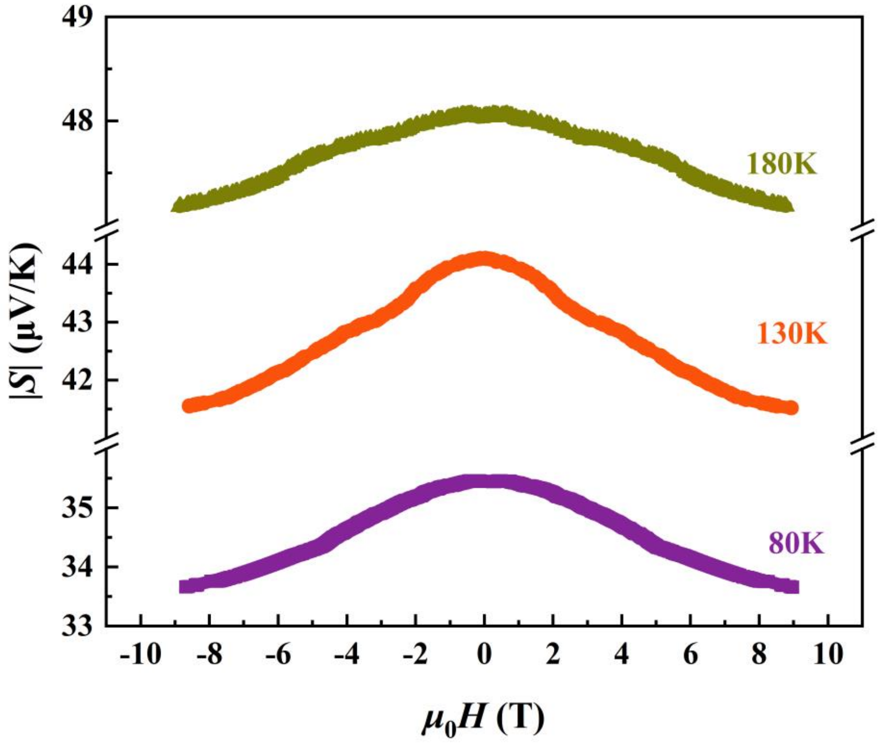

S5. Field-dependent thermopowr at 80, 130 and 180K.

Figure S5 displays the field-dependent thermopower at 80, 130 and 180 K. All the three curves show suppression of S by magnetic field which is consistent with the temperature-dependent thermopower measured under 0 and 9 T. The curve measured at 130 K close to T_c exhibits the largest suppression compared to the other two curves.

Part VI. Magnetic field induced difference temperature-dependent resistivity.

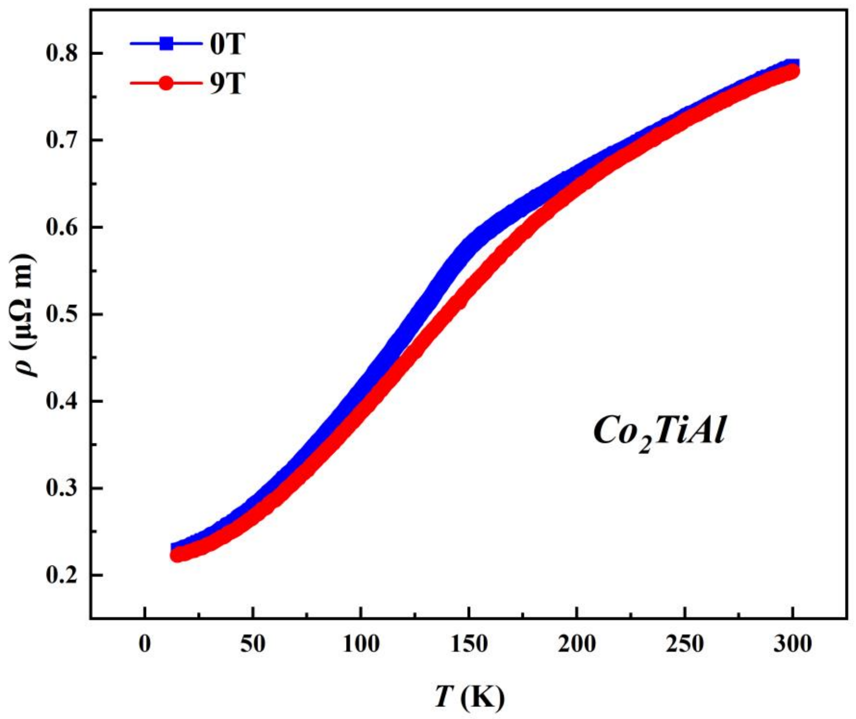

S6. Temperature-dependent resistivity under 0 and 9 T of Co_2TiAl.

Figure S6 displays the temperature-dependent resistivity of Co_2TiAl at 0 and 9 T. The blue line shows a distinct kink around the Cuire temperature at 0 T, while the red line shows a relatively smooth change at 9 T.

Part VII. Spin fluctuation induced thermopower of Co_2TiAl.

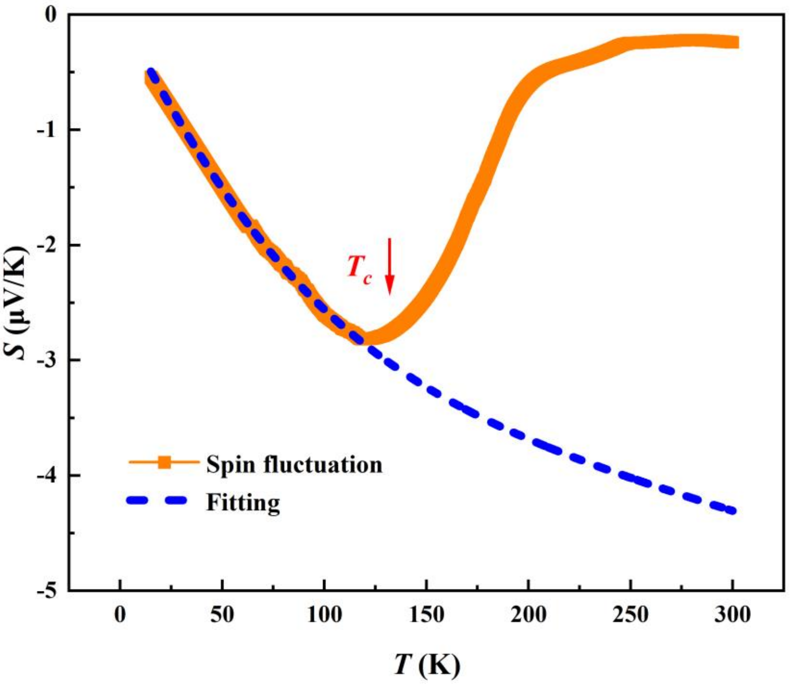

S7. Fitting and experimental results of the thermopower from spin fluctuation.

Figure S7 shows the spin fluctuation induced thermopower of Co_2TiAl. The orange solid line represents the experimental data of thermopower due to spin fluctuation. The blue dashed line represents the fitting curves of spin fluctuation induced thermopower according to formula[1] $S(\mathrm{T}) = \alpha \mathrm{T} + \beta \mathrm{T} \left(\frac{\mathrm{T}}{\bar{T}}\right)^2 \log \frac{\delta + (\mathrm{T}/\bar{T})^2}{(\mathrm{T}/\bar{\mathrm{T}})^2}$. The arrow denotes the magnetic transition temperature, and the data fit well with the theoretical result below the magnetic transition temperature.

Part VIII. Electron diffusion thermopower of Co_2TiAl

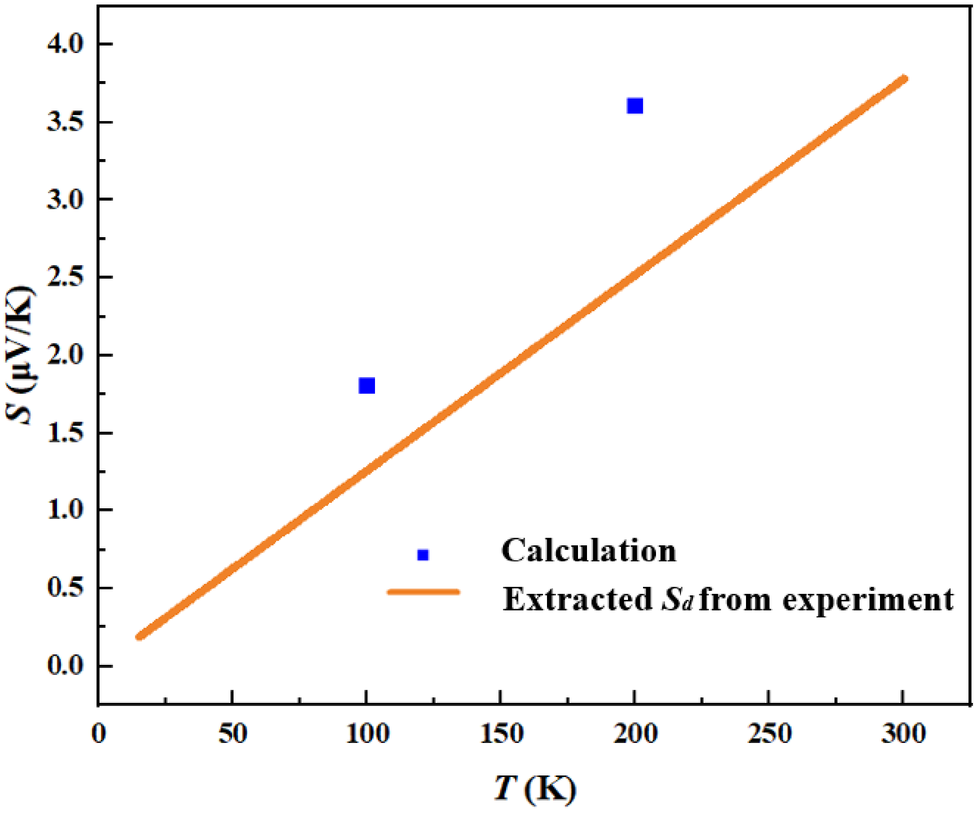

S8. Calculation and experimental results of the electron diffusion thermopower of Co_2TiAl.

Figure S8 displays the calculation and experimental results of the electron diffusion thermopower of Co_2TiAl. The calculation is based on the theoretical model of metals[2] $S_d=\frac{2}{3}(\frac{\pi}{3})^{2/3}\frac{k_B}{e}\frac{m^*}{\hbar^2}\frac{k_BT}{n^{2/3}}$, where m^* is calculated by $m^* = ne^2\tau/\sigma$ according different τ/σ reported[3] before. Here, m^* is 4.5 m_e (mass of electron)[3] and n is 6.79×10^{22} cm^{-3} at 100 K and m^* is 2.3 m_e and n is 1.97×10^{22} cm^{-3} at 200 K. The experimental result is extracted from the Seebeck coefficient at 9 T above 240 K by linear fitting. The experimental result is coincident with the calculation considering the inaccuracy of the estimation of relaxation time τ[3].

Part IX. Log-log plots for thermopower from magnon drag.

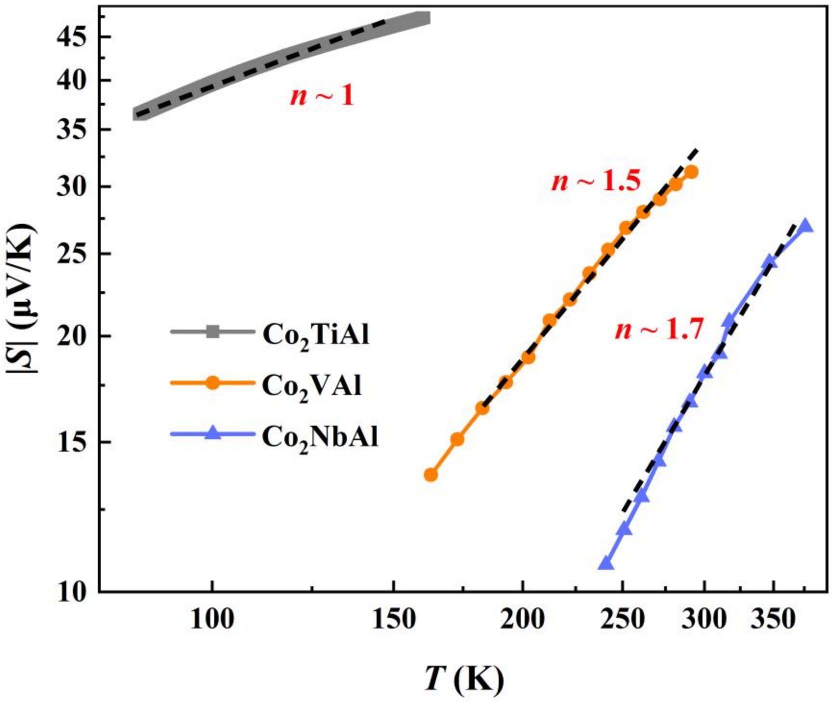

S9. Log-log plots for thermopower from magnon drag of Co_2XAl.

Figure S9 displays log-log plots for thermopower of Co_2TiAl, Co_2VAl and Co_2NbAl. The fitting data of Co_2TiAl is $|S_M|$ and the data of Co_2VAl and Co_2NbAl is $|S_M|$ approximately ignoring S_{sf} item. The data was fitted based on $S_M = \frac{2}{3}\frac{C_m}{n_e e}\frac{1}{1+\tau_{em}/\tau_m}$ [2] $\sim T^n$ regarded τ_m and τ_{em} as constants roughly.

Part X. Thermopower contribution from magnetism of Co_2VAl and Co_2NbAl.

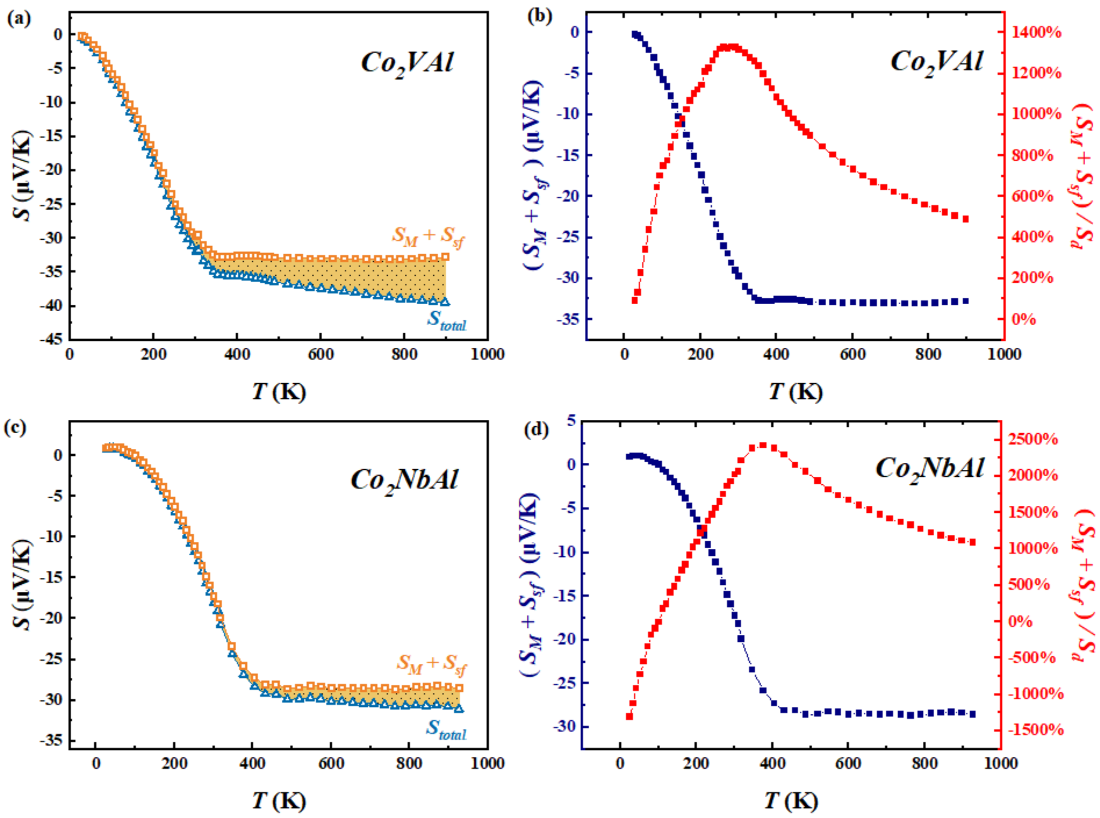

S10. Comparison between total thermopower and thermopower from magnetism of Co_2VAl and Co_2NbAl.

Figure S10 shows thermopower contribution from magnetism of Co_2VAl and Co_2NbAl. Figure S8a and Figure S8c display measured thermopower S_{total} (triangle) and thermopower from magnetism $S_M + S_{sf}$ (square) of Co_2VAl and Co_2NbAl. The blue lines in Figure S8b and Figure S8d directly shows the thermopower contribution from magnetism $S_M + S_{sf}$, and the red lines in Figure S8b and Figure S8d shows the ratios of $S_M + S_{sf}$ and S_d, which reach the maximum value above 1000% of both Co_2VAl and Co_2NbAl.

References

[1] T. Okabe, *J. Phys.-Condes. Matter* **2010**, 22, 10.

[2] S. J. Watzman, R. A. Duine, Y. Tserkovnyak, S. R. Boona, H. Jin, A. Prakash, Y. H. Zheng, J. P. Heremans, *Phys. Rev. B* **2016**, 94, 9.

[3] R. S. Sunmonu, J. O. Akinlami, E. O. Dare, G. A. Adebayo, *Materials Science and Engineering B-Advanced Functional Solid-State Materials* **2020**, 262.
